# The effect of watchful waiting compared to immediate test ordering instructions on general practitioners' blood test ordering behaviour for patients with unexplained complaints; a randomized clinical trial (ISRCTN55755886)

**DOI:** 10.1186/1748-5908-7-29

**Published:** 2012-04-04

**Authors:** Marloes A van Bokhoven, Hèlen Koch, Trudy van der Weijden, Anuska HM Weekers-Muyres, Patrick JE Bindels, Richard PTM Grol, Geert-Jan Dinant

**Affiliations:** 1 Maastricht University, CAPHRI School for Public Health and Primary care, Department of General Practice, PO Box 616, 6200, MD Maastricht, The Netherlands; 2 Dutch College of General Practitioners, PO Box 3231, 3502, GE Utrecht, The Netherlands; 3 Erasmus MC, Department of General Practice, PO Box 2040, 3000, CA Rotterdam, The Netherlands; 4 IQ Scientific Institute for Quality of Health Care, Nijmegen University, PO Box 9101, 114, 6500, HB Nijmegen, The Netherlands

## Abstract

**Background:**

Immediate blood testing for patients presenting with unexplained complaints in family practice is superfluous from a diagnostic point of view. However, many general pracitioners (GPs) order tests immediately. Watchful waiting reduces the number of patients to be tested and the number of false-positive results. The objectives of this study are: to determine the feasibility of watchful waiting compared to immediate test ordering; to determine if a special quality improvement strategy can improve this feasibility; and to determine if watchful waiting leads to testing at a later time.

**Methods:**

The study is a cluster-randomized clinical trial with three groups, on blood test ordering strategies in patients with unexplained complaints. GPs in group one were instructed to order tests immediately and GPs in group two to apply a watchful waiting approach. GPs in group three received the same instruction as group two, but they were supported by a systematically designed quality improvement strategy. A total of 498 patients with unexplained complaints from 63 practices of Dutch GPs participated. We measured: the percentage of patients for whom tests were ordered and number of tests ordered at the first consultation; performance on the strategy's performance objectives (i.e., ordering fewer tests and specific communication skills); the number of tests ordered after four weeks; and GP and patient characteristics.

**Results:**

Immediate test ordering proved feasible in 92% of the patients; watchful waiting in 86% and 84%, respectively, for groups two and three. The two watchful waiting groups did not differ significantly in the achievement of any of the performance objectives. Of the patients who returned after four weeks, none from group one and six from the two watchful waiting groups had tests ordered for them.

**Conclusions:**

Watchful waiting is a feasible approach. It does not lead to testing immediately afterwards. Furthermore, watchful waiting was not improved by the quality improvement strategy.

**Trial registration:**

Clinical trial registration: ISRCTN55755886

## Background

'Unexplained complaints in general practice can be defined as those complaints for which a general practitioner (GP), after clarifying the reason for encounter, taking the patient's history and performing physical examination, is unable to establish a diagnosis [1]. On average, 3% to 39% of consultations involve complaints considered unexplained by the GP [2-4]. The diagnostic workup for these patients frequently involves ordering blood tests. In previous research with the same inclusion criteria as used in the study we present here, we found that tests were ordered in 59% of the patients presenting with unexplained complaints [5]. However, the diagnostic accuracy of these tests is limited due to the relatively low probability of somatic pathology: less than 5% according to a rough estimate [6]. Due to false-positive results, this behaviour may even result in unnecessary further testing, leading to undesirable effects such as patient anxiety, somatisation, or high costs.

Since most unexplained complaints are self-limiting [1,7], a four-week watchful waiting approach is expected to reduce both the number of patients to be tested and the risk of false-positive test results. However, many GPs perceive barriers against watchful waiting. Some have different testing routines, regard immediate test ordering as efficient when working under time pressure, or have a low tolerance of uncertainty. Others perceive pressure from patients to order laboratory testing (even if the patient does not explicitly ask for it) or mention tactical motives for test ordering, e.g., the prevention of hospital referral or more expensive tests [8,9]. Thus, though watchful waiting appears to be a sound principle from a diagnostic point of view, immediate test ordering seems to be more attractive to both GPs and patients.

We designed a quality improvement strategy to promote the watchful waiting approach (see Contents of quality improvement strategy). The strategy was developed systematically, tailored to the barriers and facilitators perceived by GPs, and resulted in specific, consultation-related performance objectives for the GPs [10,11].

### Contents of quality improvement strategy

#### Small group meeting 1 (duration 2.5 hours)

Part 1: Interactive explanation of diagnostic value of tests for unexplained complaints and effect of watchful waiting policy on diagnostic value.

Part 2: Discussion of difficulties experienced in practice when dealing with patients presenting with unexplained complaints.

Goal setting to change behaviour in GPs' own practice.

#### Small group meeting 2 (duration 2.5 hours)

Part 1: Discussion about experiences with behaviour change. Searching for solutions to barriers that have arisen002EM

Part 2: Practicing difficult situations by means of video vignettes.

Setting new goals to change their own behaviour.

#### Practice visit (duration approximately one hour per practice)

Discussing barriers to change perceived by individual GPs and providing suggestions to overcome these, based on stage of change.

Prior to each meeting, GPs received homework assignments to prepare themselves for the meetings. In between meetings, GPs get the opportunity to work on their goals to change their behaviour.

In this study, we compared the feasibility of two approaches--watchful waiting and immediate testing--by addressing three questions. First, what is the feasibility of a watchful waiting approach compared to that of an immediate test ordering approach? Second, can the systematically developed quality improvement strategy improve performance in terms of the objectives of the watchful waiting approach? Third, what percentage of patients is tested after an initial watchful waiting period?

The study was part of a cluster-randomized clinical trial in which the instruction to apply a watchful waiting approach, with or without the support of the quality improvement strategy, was compared with the instruction to order blood tests immediately [12].

## Methods

### Design

The full protocol of this cluster-randomized trial has been published elsewhere [11,12]. To prevent contamination through patients and individual GPs, the GPs were randomized at practice level. Practices were randomized over three groups using a random number seed computer program for block randomization. Group one was instructed to order blood tests immediately, groups two and three to apply a four-week watchful waiting approach. Only group three was supported by our systematically developed quality improvement strategy.

The medical ethics review boards of both the Academic Medical Center-University of Amsterdam and the University Hospital Maastricht approved the study.

### Participants

#### General practitioners

For logistic reasons, regional laboratories in the western and southern regions of the Netherlands were asked to participate first. All GPs using the facilities of these regional laboratories were asked to participate in the trial.

### Patients

The GPs were asked to enroll each consecutive eligible patient. Patients aged 18 years and older were eligible for participation if they presented with one of the following complaints: fatigue, abdominal complaints, weight changes, musculoskeletal complaints, or itch. Their complaints needed to be unexplained according to the definition given in the Background section above. Patients also had to be able to read, speak, and understand Dutch. Excluded were patients with unexplained complaints that caused a sense of alarm in the GP, making watchful waiting unacceptable. Patients were instructed to re-consult if their complaints had not resolved after four weeks. In the Dutch health care system, patients are registered in a practice (list system) and GPs have a gate-keeping role. This means that patients usually do not visit other GPs without referral by their GP. The patients were given written information by the GP and were asked to give informed consent. We kept patients in the watchful waiting groups naive about the possibility of getting blood tests ordered to prevent bias. In our opinion, this was ethically acceptable because both diagnostic approaches are usual care. Patients in the immediate test ordering group were fully informed about both diagnostic approaches.

### Quality improvement strategy

The development of the strategy and its contents have been described elsewhere [11,12]. It consisted of two small group sessions and one practice outreach visit, whose contents have been summarized in "Contents of quality improvement strategy". The performance objectives (Table 1, first column) were communicated to the GPs of group three during all these contacts.

**Table 1:** Scores on performance objectives for GPs (1st column) and operationalisation to measure the performance (2nd column)

Performance objective	Operationalisation of performance objective	Answering categories	Group one (immediate test ordering) n = 229 patients	Group two (watchful waiting) n = 95 patients	Group three (watchful waiting + quality improvement strategy) n = 174 patients
	Patients for whom tests were ordered n (%)		210 (91.7)	13 (13.7)	27 (15.5)

Ordering fewer tests at the same time	Mean number of tests ordered (SD)		7 (3.7)	7 (2.1)	6 (2.6)

Performing adequate history taking and physical examination	GP performed physical examination % (n)	Sufficient Not sufficient	124 (56.9) 94 (43.1)	57 (63.3) 33 (36.7)	99 (62.7) 59 (37.3)

Explaining findings to the patient	Patient understood GP's explanation of the complaints n (%)	At least sufficient Insufficient/unknown	184 (84.4) 34 (15.6)	79 (90.8) 8 (9.2)	139 (88.5) 18 (11.5)

Explaining that findings are currently not explained by specific diseases	Patient understood seriousness of complaints after the consultation n (%)	Yes No	55 (25.5) 161 (74.5)	34 (37.8) 56 (62.2)	57 (36.5) 99 (63.5)

Discussing four-week watchful waiting approach with patient	GP discussed the possibilities of additional tests with the patient n (%)	Sufficient Insufficient/unknown	153 (70.2) 65 (29.8)	54 (60.7) 35 (39.3)	81 (51.3) 77 (48.7)

Asking the patient to return if the complaints do not resolve in a month	GP asked the patient to return if the complaints did not disappear n (%)	Yes No/unknown	145 (67.4) 70 (32.6)	80 (88.9) 10 (11.1)	131 (82.9) 27 (17.1)

### Variables and measurements

The primary outcome variables were the percentage of patients for whom tests were ordered and the number of tests ordered at the first consultation. Secondary outcome variables were the GPs' performance in terms of the performance objectives and the numbers of tests ordered when the patient returned after approximately four weeks. Explanatory variables were GP and patient characteristics.

When laboratory tests were ordered by the GP, either at the first consultation or when the patient returned after approximately four weeks, the research team received a copy of the test results form. The GPs were asked to order a pre-specified set of tests for all patients. In addition, GPs were asked which tests they would have ordered themselves. They were also given the option of stating that they would not have ordered any tests outside the research setting.

The GPs' performance was measured in two ways: by asking the GPs to record their own performance and by asking the patients to report their experiences with regard to their GPs' behaviour (see Table 1, second column). At the patients' first visit, GPs filled in a complaint registration form. Each patient received a patient questionnaire with questions about their background characteristics, what happened during the consultation, and their levels of satisfaction and anxiety. The questionnaire was handed out to them by the GPs at the end of the first consultation, with an envelope in which they could return the questionnaire to the research team immediately after filling it in at home.

The explanatory variables were assessed at the start of the research project by having all GPs fill in a background characteristics form. Data were collected over a period of two years.

### Analysis

Except for randomization, variables were dichotomized, using the mean when appropriate (Table 1). Watchful waiting with and without quality improvement strategy (groups three and two, respectively) were compared with the immediate test ordering approach (group one). Subsequently, groups two and three were compared. The percentage of patients for whom blood tests were requested immediately was first determined per randomization group. When GPs indicated that they would have ordered tests themselves, we also analyzed the number of tests they would have ordered per consultation. Practical limitations meant that we could only analyze this for the period of one year. Subsequently, we used a bivariate analysis to evaluate if the GPs in group three had met the performance objectives regarding consultation skills, compared to groups one and two. We did not account for clustering of patients within GP practices because we know from a different study among the same patients that the intra-cluster correlation coefficient was extremely low (2.37 e^-7^) [13]. Finally, we analyzed the percentage of patients who had been tested after an initial watchful waiting period. All analyses used the SPSS 11 statistical software package.

## Results

### Participant flow and background variables

**Figure 1:**
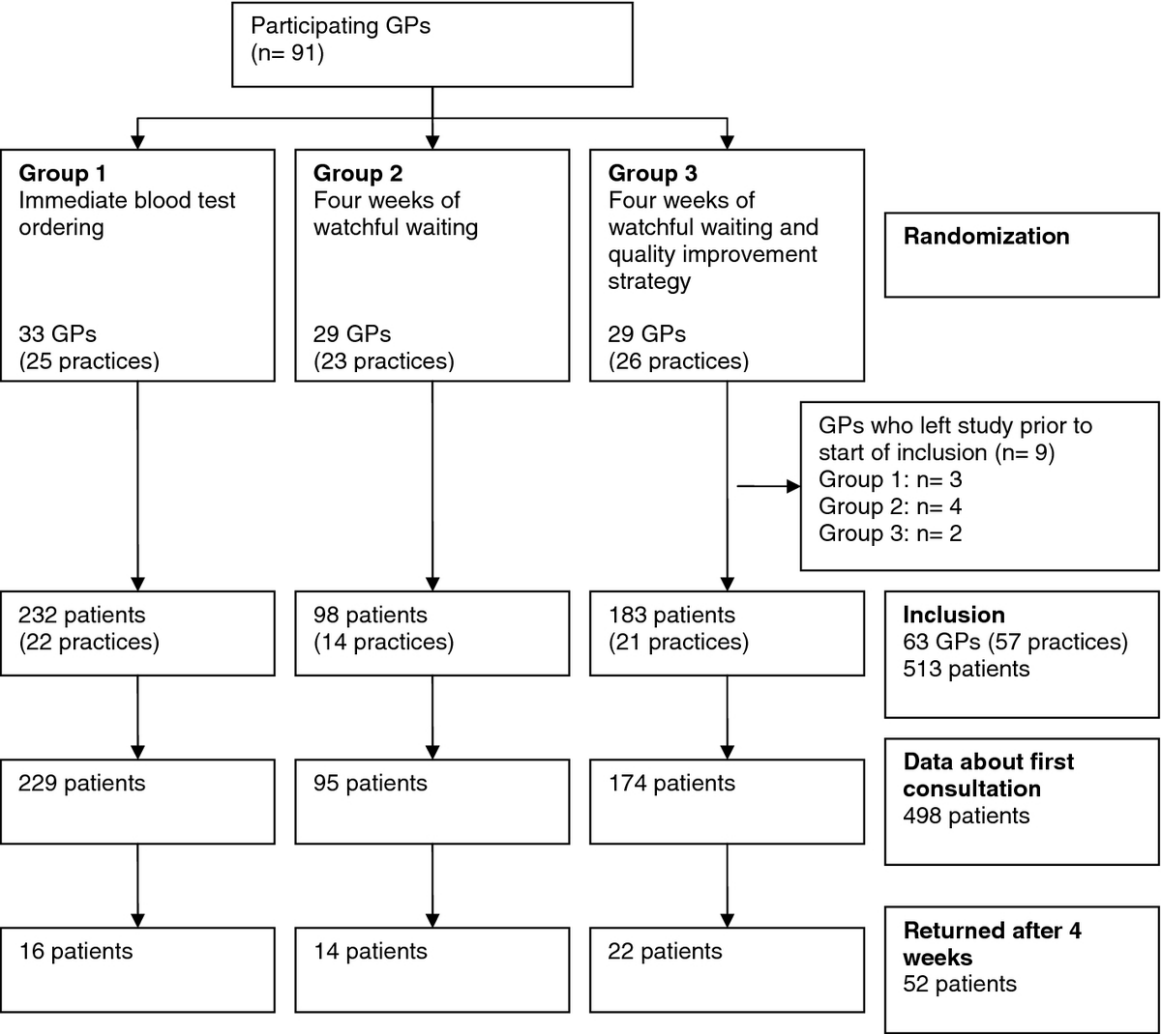
**Participant flow**.

Inclusion took place from February 2002 until December 2003. In total, 91 GPs were randomized in the study, nine of whom ended their cooperation before the inclusion started, mainly due to private circumstances and pressure of work, and 19 did not include any patients. As a result, 63 GPs in 57 practices included 513 patients. Data about the first consultation were available for 498 patients (Figure 1). Fifty-two patients returned after approximately four weeks.

Background data of both GPs and patients are summarized in Tables 2 and 3. Of the 63 GPs who included patients, 27 were in group one (22 practices), 14 in group two (14 practices), and 22 in group three (21 practices). The mean age of the participating GPs was 45 years, and 74% of them were male. The mean number of years of experience was 13. The mean age of the patients was 43, and 28% were male. None of the differences between the randomization groups were statistically significant (*p *> 0.05).

**Table 2:** Background data of participating GPs (n = 91)

Variables	Categories	Results overall n = 91 GPs	Randomization		
			
			Immediate n = 33 GPs	Watchful waiting n = 29 GPs	Watchful waiting + strategy n = 29 GPs
Mean age, years (SD)		45 (7.3)	47 (5.8)	44 (7.2)	45 (8.8)

Gender n (%)	Male	67 (74)	26 (79)	17 (59)	24 (83)

Mean number of years of experience as a GP (SD)		13 (8.7)	14 (7.1)	11 (8.4)	14 (10.4)

Practice type (soloists versus other)n (%)	Solo	34 (37)	9 (27)	15 (52)	10 (34)

Practice location n (%)	Urban Semi-rural Rural	45 (50) 11 (12) 34 (38)	15 (47) 4 (13) 13 (41)	15 (52) 5 (17) 9 (31)	15 (47) 4 (13) 13 (41)

**Table 3:** Background data of participating patients (n = 498)

Variables	Categories	Results overall (n = 498)	Randomization^#^		
			
			Immediate (n = 229)	Watchful waiting (n = 95)	Watchful waiting + strategy (n = 174)
Mean age in years (SD)		43 (16.2)	42 (15.5)	45 (15.2)	45 (17.5)

Gender (%)	Male	140 (28)	67 (29)	18 (19)	55 (32)

Type of health insurance: private versus state (%)	Private	164 (33)	80 (35)	37 (39)	47 (27)

Highest level of education (%)	None completed Primary Secondary Higher	4 (1) 46 (10) 323 (67) 106 (22)	2 (1) 20 (9) 146 (64) 55 (24)	1 (0) 5 (1) 65 (68) 21 22)	1 (0) 21 (12) 112 (64) 30 (17)

^#^Differences between groups were statistically tested with *X*^2 ^tests or *t*-test when appropriate. No statistically significant differences (*p *> 0.05) were found between the randomization groups

### Feasibility of watchful waiting

The effects of the interventions have been summarized in Table 1. The watchful waiting approach was applied to 82/95 patients of group two (86%) and 147/174 of group three (84%), whereas 210/229 patients in group one (92%) were tested immediately. There was no statistically significant difference between the two watchful waiting groups in terms of the number of patients for whom tests were ordered (odds ratio (OR) 0.86, 95% CI 0.42 to 1.76).

### Performance objectives

Groups two and three did not differ significantly as regards meeting any of the performance objectives (Table 4). A comparison of watchful waiting with immediate test ordering showed that there were no significant differences between group one versus groups two and three in terms of the performance objectives 'GP orders fewer tests,' 'GP performs adequate physical examination,' and 'GP explains findings to patient.' When compared to group one, the GPs in group three had lower scores on the item 'GP discusses the value of additional tests' (OR 0.4 95%; CI 0.3 to 0.7)). GPs in the watchful waiting groups had better scores for knowledge about the seriousness of the complaints and for the objective 'GP asks the patient to return if the complaints do not disappear within a month' (Table 4).

**Table 4:** Odds ratios (confidence intervals) of differences in meeting performance objectives between groups

Operationalisation of performance objective	Group two versus group one	Group three versus group one	Group two versus group three
Patients with tests ordered	N/A	N/A	N/A

Mean number of tests ordered	N/A	N/A	N/A

GP performed physical examination	1.3 (0.8 to 2.2)	1.3 (0.8 to 1.9)	1.0 (0.6 to 1.8)

Patient understood GP's explanation of the complaints n (%)	1.8 (0.8 to 4.1)	1.4 (0.8 to 2.6)	1.3 (0.5 to 3.1)

Patient understood seriousness of complaints after the consultation	1.8 (1.1 to 3.0)*	1.7 (1.1 to 2.6)*	1.1 (0.6 to 1.8)

GP discussed the possibilities of additional tests with the patient	0.7 (0.4 to 1.1)	0.4 (0.3 to 0.7)*	1.5 (0.9 to 2.5)

GP asked the patient to return if the complaints did not disappear	3.8 (1.9 to 7.9)*	2.3 (1.4 to 3.9)*	1.6 (0.8 to 3.6)

### Testing after watchful waiting

At the first consultation, GPs ordered a mean of seven tests in groups one and two, and six in group three. After the watchful waiting period of approximately four weeks, GPs in group one would not have ordered tests themselves for any patient. In group two, one patient had six tests ordered for them, and in group three, five patients had a mean of nine tests ordered for them.

## Discussion

Our findings show that watchful waiting is a feasible approach for patients presenting with unexplained complaints in general practice. However, our quality improvement strategy did not improve the feasibility of a watchful waiting approach, nor did it improve the GPs' consultation skills. After an initial watchful waiting period, laboratory testing was rarely used later on. This was predominantly due to the fact that not many patients returned. The instruction to apply a watchful waiting approach, with or without additional training, was thus an effective way to reduce test ordering.

The lack of effect of the quality improvement strategy might be explained by the fact that the room for improvement was very limited, given the good feasibility of watchful waiting even without GPs being exposed to the quality improvement strategy. On the other hand, the strategy may not have had the intended effect. Given the positive attitude regarding immediate test ordering and lack of perceived disadvantages, the GPs may have lacked a sense of urgency to change their blood test ordering behaviour [8]. When we design a new quality improvement strategy to improve GPs' test ordering behaviour we will need to place greater emphasis on the negative effects of superfluous testing. This may be done by making visible the implications of cascade effects on patients' well being. Another possibility, however, is that more intensive training is needed, given the complexity of the skills that need to be applied.

In terms of consultation skills, it appears that GPs who apply a watchful waiting approach replace testing by providing patients with explanations about the complaints. This can be considered a positive effect because it may favourably influence the patients' satisfaction and decrease anxiety [14,15]. The value of additional tests was more frequently discussed in group one than in groups two and three. However, it is questionable whether the GPs in group one discussed the advantages of a watchful waiting approach, including the limitations of laboratory tests, as was intended by the performance objective. The difference between the groups appears to have been due to the different wording of the performance objective used in the patient questionnaire ('GP discussed the possibilities of additional tests with the patient'), because we wanted to blind the patients to the specific test ordering strategies we wanted to study. Finally, GPs in the watchful waiting groups more frequently asked patients to return if the complaints should persist than those in group one.

The behaviour of group one may have an important disadvantage. Unspecified testing carries not only the risk of false-positive tests, but also that of false-negative results. In the literature, it has been repeatedly suggested that patients may be incorrectly reassured by negative test results and consequently may not return even if their complaints persist, which may cause a diagnostic delay. Empirical evidence is limited, however [16-20].

One strength of the present study was that, as far as we know, it represents the first time that a watchful waiting approach has been studied as a diagnostic strategy. So far, the approach of delaying further action has only been described in studies on therapy and monitoring. An example of its use in therapy is the prescription of antibiotics with the instruction only to start taking them when the complaints last for a specified time or become severe enough. This is called 'delayed prescribing' [21,22]. An example of the use in monitoring, which is usually also described by the term 'watchful waiting' is to monitor the levels of prostate-specific antigen in patients with prostate carcinoma. Only when they rise to a certain level are therapeutic interventions started.

Another strength of this study was that our systematically developed quality improvement strategy was also systematically evaluated by taking into account the performance objectives as intermediate outcome measures.

A limitation of the study was that selective patient inclusion may have caused bias. The GPs of group two (watchful waiting without quality improvement strategy) included fewer patients in the study than those of other groups. This seems to have been due to a number of GPs in this group who did not include any patients; if these GPs are omitted from the analysis, no statistically significant difference in patient inclusion remains between the groups. Our explanation is that GPs hesitated to start asking patients to participate because they did not have any diagnostic tests to offer them and that could serve as a 'reward' for participation. If this was indeed the case, GPs in groups two and three should have included fewer patients. However, GPs in group three had participated in several training sessions in which they discussed the limited value of immediate test ordering and the effects of watchful waiting. Therefore, GPs in group three may have felt more confident about convincing patients to participate. To prevent selective inclusion, the GPs were allowed to order tests immediately if they felt it would be wrong to postpone testing, and they were asked to explain their reasons on a special form. They mentioned three types of reasons: their own sense of alarm (n = 10), the requests by patients or their relatives to have tests done (n = 7), and the findings from a patient's background, history, and physical examination (n = 7). Although we have no indications of selective inclusion, we cannot completely exclude it either. A non-inclusion analysis was not possible because GPs did not register unexplained complaints in the patient records.

Another limitation was that we only evaluated delayed blood test ordering immediately after the watchful waiting period, while it is known from other studies that unexplained complaints tend to persist longer [23,24]. However, many of these patients do not return to their GPs because they find a way to manage their complaints themselves [23]. Further research is necessary to determine if watchful waiting induces delayed testing at a later time.

A point of discussion is whether or not these findings can be generalised to general practices in other countries. In the Netherlands, each patient is listed to one GP practice. This ensures continuity of care and creates a basis for trust. In countries where patients see different doctors at each visit or easily 'shop around' among doctors, it might be more difficult to implement a watchful waiting approach. Furthermore, the GPs' tolerance of uncertainty differs between countries. It is known that this may influence the GPs' patient management behaviour [25]. This limited tolerance is sometimes caused by fears of malpractice lawsuits.

## Conclusions

This study shows that watchful waiting is a feasible approach in patients with unexplained complaints, and that it does not lead to delayed testing within the first six weeks. However, the diagnostic value of immediate testing compared to the watchful waiting approach needs to be taken into account when drawing definitive conclusions on the desirability of watchful waiting. Further research is needed to determine if the approach will actually be used in daily practice routine, in which the advantages of immediate test ordering in the interaction with patients may outweigh the advantages of evidence-based, test-ordering behaviour. Given the high level of feasibility found in the present experimental setting, further research on watchful waiting is warranted.

## Competing interests

The authors declare that they have no competing interests.

## Authors' contributions

MAB co-developed the measurement instruments, did the data collection in the southern part, analysed the data, and wrote the first draft of the manuscript. HK co-developed the measurement instruments, did the data collection in the northern part, and critically revised the manuscript. TW co-developed the protocol, participated in data analysis, and critically revised the manuscript. AWM participated in data-collection and analysis and critically revised the manuscript. PB was involved in operationalising the protocol, development of the measurement instruments, and data collection. He critically revised the manuscript. RG co-developed the protocol and critically revised the manuscript. GJD co-developed the protocol, supervised the project, participated in data analysis, and critically revised the manuscript. All authors read and approved the final manuscript

## Query

1. Article title: Title in the manuscript "The effect of watchful waiting compared to immediate test ordering instructions on general practitioners' blood test ordering behaviour for patients with unexplained complaints; a randomized clinical trial (ISRCTN55755886)" differs from the jobsheet "The effect of watchful waiting instructions on GPs' blood test ordering behaviour for patients with unexplained complaints; a randomized clinical trial (ISRCTN55755886)". We have proceeded and followed the manuscript. Please check and advise if action taken is appropriate.

2. Abstract: As per journal standards the following headings are required within the 'Abstract': Background; Results; Conclusions. However extra headings "Methods" have been included. Please alter where appropriate.

3. Abbreviations: If abbreviations are used in the text they should be defined in the text at first use, and a list of abbreviations can be provided, which should precede the competing interests and authors' contributions. However, list of abbreviations were not provided. Please supply the abbreviation list. Otherwise, kindly advise us on how to proceed.

4. Tables: Journal requires that the first table referenced in the manuscript text should be Table 1. The second, Table 2, etc. However, original sequence of the table citations "Tables 2, 3, 4, 5" are out of order. Tables and citations were reordered so that they are cited in consecutive order. Please check if action taken is appropriate. Otherwise, kindly advise us on how to proceed.

5. Tables: Please specify the significance of footnote (*) cited in Table 4, as a corresponding footnote text has not been provided.
